# Exploring Barriers to Accessing Mental Health Services for Adolescents With Depression in Pokhara Metropolitan City, Nepal: Perspectives of Mental Healthcare Providers

**DOI:** 10.7759/cureus.117755

**Published:** 2026-10-11

**Authors:** Mia Thompson, Alastair Cardno, Deepak Joshi

**Affiliations:** 1 Nuffield Centre for International Health and Development, University of Leeds, Leeds, GBR; 2 Division of Psychological and Social Medicine, University of Leeds, Leeds, GBR; 3 Department of Research and Development, HERD International, Kathmandu, NPL

**Keywords:** adolescents, barriers, depression, mental health, mental health services, nepal

## Abstract

Background

In Nepal, depression is one of the most common mental health conditions among adolescents, but mental health services for this population remain limited. Previous studies have identified barriers to accessing mental health services in Nepal; however, to our knowledge, no study has specifically examined the barriers faced by adolescents with depression.

Aims

This study aimed to identify barriers to accessing mental health services for adolescents with depression in Pokhara Metropolitan City, Nepal, from the perspectives of mental healthcare providers, and to identify approaches to overcome these barriers.

Method

Semi-structured interviews were conducted with nine healthcare professionals involved in delivering mental healthcare, including counsellors, psychologists, medical officers, and consultant psychiatrists. Participants were selected through purposive sampling according to predefined inclusion and exclusion criteria. Interviews were conducted in May 2023 at the participants’ workplaces across Pokhara Metropolitan City. The interviews were audio-recorded and transcribed verbatim. Reflexive thematic analysis was used to develop codes and themes.

Results

Barriers to accessing mental health services included individual factors, primarily related to limited knowledge of mental health and available services; social factors, including stigma, particularly within families and the wider community; and systemic and structural factors, including limited service availability and an insufficient mental health workforce. Suggested strategies to overcome these barriers included raising mental health awareness in schools and through social media, as well as increasing the number of mental health-trained staff providing services within communities.

Conclusions

Recommendations for future practice include integrating mental health education into Nepal’s national school curriculum and expanding training programs for healthcare workers. Future research could explore perspectives from other regions of Nepal and include additional stakeholder groups, such as adolescents, parents of adolescents, and government staff.

## Introduction

Adolescence is the phase between childhood and adulthood, commonly defined as occurring between the ages of 10 and 19 years [1]. Physical, psychological, and behavioral changes during this period may increase adolescents’ vulnerability to mental health issues [2]. Mental health is a state of well-being that encompasses an individual's feelings, thoughts, and emotions [3]. Globally, one in seven adolescents experiences a mental health condition [2]. These conditions include mental disorders and psychosocial disabilities that cause distress and impair functioning [3], with depression being one of the most common mental health conditions among adolescents [2]. Depression is a mood disorder characterized by feelings of sadness, irritability, or emptiness; loss of pleasure in activities; and frequent disturbances in sleep and appetite [4]. It is also a major risk factor for suicide, which is the third leading cause of death among people aged 15-29 years. Low- and middle-income countries (LMICs) account for 73% of global suicides [5]. However, the actual figures may be higher because adolescent suicide is often underreported to protect families from the associated stigma [6]. Mental health is influenced by a range of factors, including the quality of home life, relationships with peers, social media use, poverty, and access to education [4,7].

Depression is one of the most common mental health conditions among adolescents in Nepal [8]. Community-based studies in Nepal have found that at least 27% of high school students experience depressive symptoms [9], with some studies reporting estimates as high as 76% [10]. The disparity between these figures is likely attributable to differences in the self-report questionnaires used to assess depression, as well as variations in sample sizes and study settings. Further research is needed to establish the prevalence of depression among adolescents in Nepal and identify optimal interventions to reduce its burden.

In Nepal, mental healthcare has been incorporated into basic healthcare services, which cover conditions such as depression, anxiety, psychosis, substance abuse, and alcohol use [11]. Mental health services provide assessment, treatment, and support for people with mental health conditions [3]. In Nepal, these services are delivered through a combination of inpatient and outpatient care in medical colleges, government hospitals, and private hospitals, with services largely concentrated in urban areas [11]. More recently, non-governmental organizations (NGOs) have played a vital role in providing community-based services; however, service coverage remains limited [12].

The Government of Nepal developed the National Mental Health Strategy & Action Plan 2020-2025, which included plans to integrate mental health services into primary care and provide free treatment to improve access [13]. However, the availability and readiness of services remain limited: only one-quarter of health facilities provide mental health services, and many of these facilities lack essential medicines, guidelines, and trained staff [14]. Furthermore, the lack of a clear accountability framework and leadership within the mental health sector negatively affects the implementation of these plans [15]. The integrated delivery of mental health services is also hindered by poor coordination between levels of government and across the health, education, and social services sectors [15].

Adolescents were also identified as a key target population for mental health improvement in the 2020 action plan [13]. However, Nepal currently has only three mental health facilities specifically for adolescents and no subspecialty training programs in child and adolescent mental health [11]. Given the challenges the local health system faces in delivering basic mental health services, providing interventions tailored to adolescents remains particularly difficult. This study therefore seeks to contribute to understanding the gaps in mental health service provision for adolescents.

Study aims

Previous studies have identified barriers to accessing mental health services for adults in Nepal, including stigma; limited knowledge of mental health and available services; transportation difficulties; inadequate human resources and availability of psychotropic medicines; financial barriers; and limited staff competency [16-18]. However, to our knowledge, no study has specifically examined the challenges faced by adolescents with depression when accessing mental health services in Nepal. This study therefore aims to identify barriers to accessing mental health services for adolescents with depression in Pokhara, Nepal, and to explore approaches to overcoming these barriers from the perspectives of mental healthcare providers. This perspective is valuable because it can help illuminate systemic barriers that may not be fully captured by patient-reported data.

This article was previously presented as a meeting abstract at the Royal College of Paediatrics and Child Health Conference, held from 26 to 28 March 2025.

## Materials and methods

To ensure comprehensive reporting, the Consolidated Criteria for Reporting Qualitative Studies (COREQ) checklist was used to guide reporting [19].

Ethics

Ethical approval was granted by the Leeds Institute of Health Sciences Research Ethics Sub-Committee at the University of Leeds and the Nepal Health Research Council (approval number: 241/2023). All participants provided written informed consent.

Design

A qualitative methodology was chosen to explore participants’ perspectives and experiences of barriers to accessing services. Semi-structured interviews were considered the most suitable data collection method because they allow interviewers to ask both primary and follow-up questions flexibly, enabling a deeper understanding of participants’ perspectives and allowing them to express their views in their own words. The interview guide, provided in the Appendices, was structured around the study objectives. It was developed based on a literature review of relevant studies conducted by the author (M.T.) and discussions with colleagues at the project’s host organization, HERD International, a national research and development organization in Nepal. The interview guide was not pilot-tested before data collection due to time constraints.

Study sample

Purposive sampling was conducted by the co-author (D.J.), who worked at HERD International in Nepal and therefore had extensive knowledge of local healthcare facilities and the roles of different mental healthcare providers. This approach was used to ensure diversity among the selected healthcare providers in terms of gender, job role, work experience, and type of health facility.

Participants were identified and selected according to the following inclusion criteria: being a mental healthcare staff member; having experience delivering mental healthcare to adolescents; being at least 18 years old; and being able to provide informed consent.

The participant recruitment process is illustrated in Figure 1.

**Figure 1:**
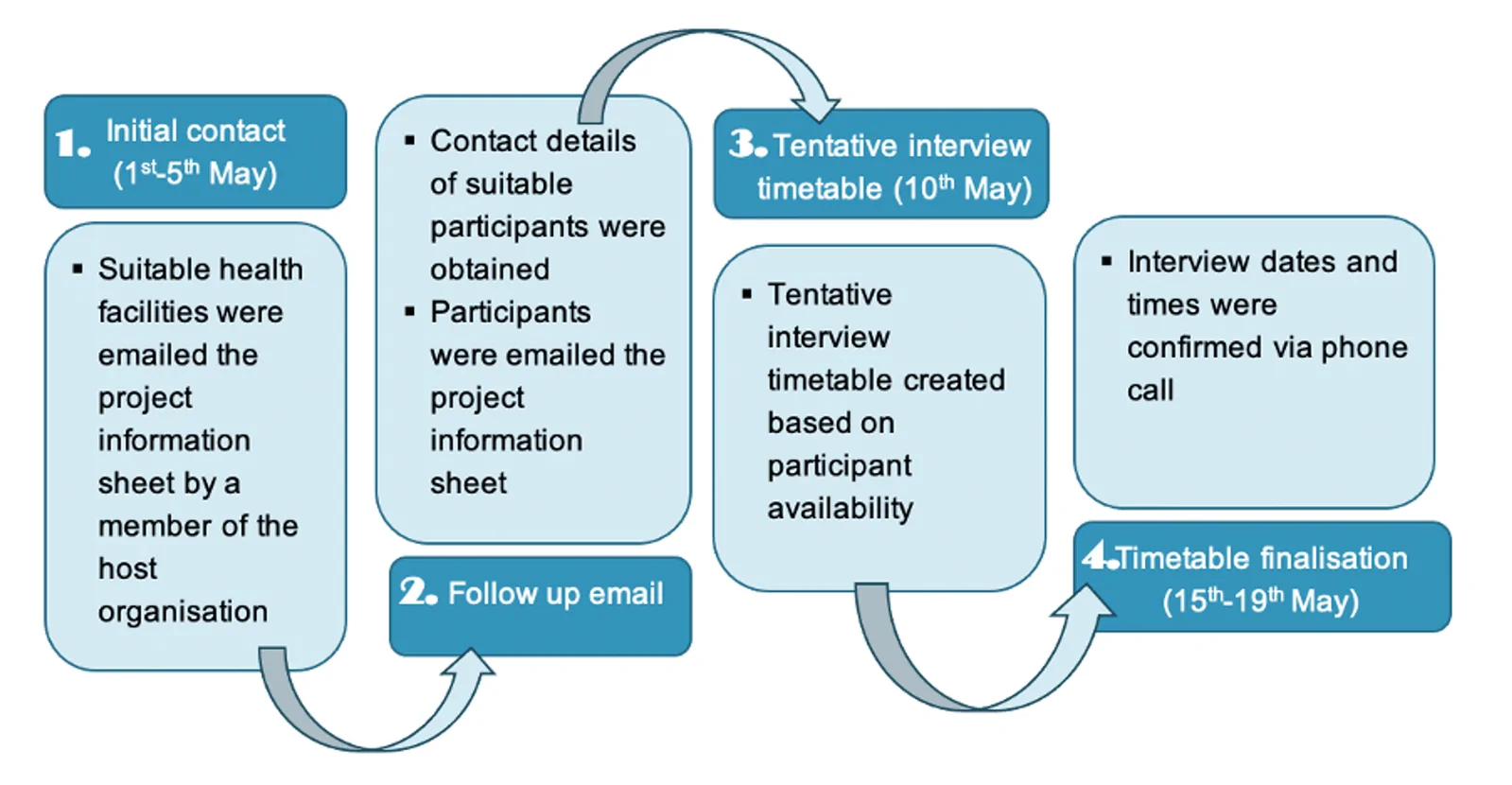
Recruitment process

As the study used reflexive thematic analysis, the adequacy of the nine-interview dataset was assessed based on the richness of the data, the diversity of participants’ experiences, the depth of the resulting themes, and the extent to which the themes addressed the research objectives.

Data collection

Interviews were conducted in May 2023 at participants’ workplaces across Pokhara Metropolitan City. This region was selected by the study’s host organization, HERD International, because of its existing knowledge of local healthcare organizations and established contacts with these organizations, which facilitated participant recruitment. Before the interviews, participants received an information sheet, and written informed consent was obtained by the research interviewer (M.T.). The researcher and participant were the only people present during five interviews, which were conducted in English. An English-Nepali interpreter was present during the other four interviews; the interpreter was either the gatekeeper or a colleague from one of the healthcare facilities. Interpreters were briefed on confidentiality before the interviews. The interview guide, provided in the Appendices, was developed in line with the study objectives to ensure that each objective was adequately addressed. Interviews lasted approximately 20-40 minutes, depending on whether an interpreter was used. With participants’ consent, interviews were audio-recorded using a Dictaphone, and notes were taken in a diary. No repeat interviews were conducted. Interviews were transcribed manually within two days of each interview to retain paralinguistic features. The audio recordings were deleted after transcription, and each transcript was assigned a code to protect participants’ confidentiality.

Data analysis

Before analysis, all interviews were transcribed in English. Reflexive thematic analysis was conducted to identify and interpret patterns of meaning across the dataset. The analysis followed six phases. Phase 1: ​​​​​​​Transcripts were read multiple times to facilitate familiarization, and initial ideas and notes were recorded. Phase 2:* ​​​​​​​*A priori codes derived from a literature review conducted by the lead researcher (M.T.) were used alongside inductive codes generated from the transcripts. Relevant sections of the transcripts were highlighted, and codes were reviewed and revised where necessary. Coding decisions and revisions were documented in an Excel spreadsheet.​​​​​​​ Phase 3: ​​​​​​​Codes with similar meanings were grouped, and potential themes and subthemes were developed. A thematic map was generated.​​​​​​​ Phase 4: ​​​​​​​Potential themes were compared against the transcripts to identify missing information and assess their fit with the dataset. The themes were then refined.​​​​​​​ Phase 5:The final themes were clearly defined and named. Phase 6: The findings were presented using the identified themes and relevant extracts from the interview transcripts.

## Results

Participant demographics and job roles are presented in Table 1.

**Table 1:** Participant demographics and job roles

Participant characteristic	Distribution
Gender	4 males
	5 females
Job role/academic qualification	4 counsellors
	2 psychologist/counsellors
	1 medical officer
	2 consultant psychiatrists
Job location	3 Koshish (non-governmental national mental health self-help organization)
	3 transcultural psychosocial organization (non-governmental organization)
	2 BG Hospital and Research Centre
	1 urban health
Duration of job role (mean, standard deviation, range)	Mean: 4 years
	Standard deviation: 4.7 years
	Range: 6 months to 16 years
Interpreter used?	4 yes
	5 no

Figure 2 presents the thematic map developed to illustrate the relationships between the identified themes and subthemes.

**Figure 2:**
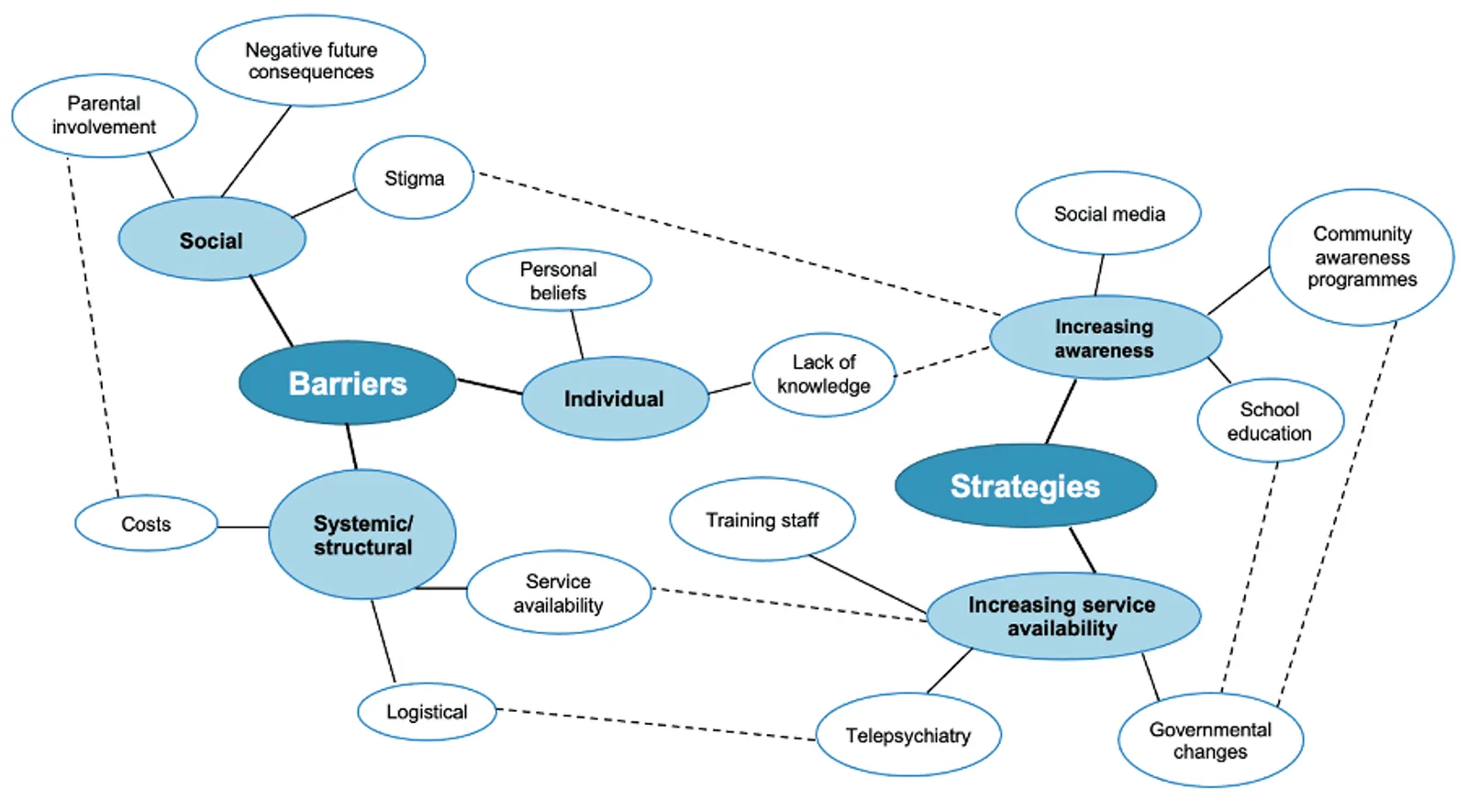
Thematic map of codes and themes

Barriers to access

Our analysis revealed three main themes for barriers to access to services, and within these, further subthemes (Table 2). 

**Table 2:** Barriers to access: themes and subthemes

Themes	Subthemes
Individual factors	Lack of knowledge
	Personal beliefs
Social factors	Stigma
	Fear of negative consequences in the future
	Parental involvement
Systemic/structural factors	Service availability
	Logistical
	Costs

Quotes from the transcripts relating to the themes of barriers to access are presented in Table 3 to illustrate the key findings. Quotes were largely retained verbatim, including grammatical features and local phrasing. However, minor edits were made where necessary to clarify meaning, with any additions or alterations indicated by square brackets within the quotations.

**Table 3:** Barriers to access according to the participants In parentheses at the end of each quote are participant gender and a three-digit number corresponding to the participant's unique identifier.

Theme	Subtheme	Quotes
Individual factors	Lack of knowledge	“So many adolescents doesn’t know about the concept of mental health, it’s not the thing that we often discuss like other physical illnesses” (102, Male)
		“They don’t know where they can get help and how to go about getting help” (103, Female)
		“They won’t think talking will do as much thing as eating medicine” (106, Female)
		“They are just calling for general asking about the doctor’s time, when they will be available, for other purposes, not for suicidal prevention” (106, Female)
		“Because of lack of awareness, those things not being included in the syllabus, the adolescents have difficulties recognising and accessing the mental health services” (103, Female)
	Personal beliefs	“People mostly go to priests and religious people to get help rather than going to medical professionals… they even change their religion to cure the problem” (101, Female)
		“Even if they have problems, firstly they don’t accept that they have the problem” (103, Female)
		“They take their problems with themselves, so they don’t want to open up” (107, Female)
Social factors	Stigma	“If somebody has diabetes…they talk about it openly and take medicines openly but when it comes to mental health issues it’s all hush hush” (103, Female)
		“They ask me where else we can meet you, and the reason being, anybody who enters this hospital for service is labelled insane” (105, Male)
		“Unfortunately, it’s not only there in common people, it’s there among the medical facility” (105, Male)
		“They want to be seen somewhere else, not in the mental health facility” (109, Male)
	Fear of negative consequences in the future	“Adolescents have to worry more about their future…and Nepali society, it’s harder to get married and even get a job when people know you have mental health issues” (101, Female)
		“They feel the losses in job, education or marriage problems so they hide” (108, Female)
	Parental involvement	“Depression cases also…they think that their children are getting lazy and not supporting in regular household jobs…they think because of the age, but they actually have mental health problem” (108, Female)
		“I know many lives lost because of parental stigma, just because they did not want to bring them for treatment” (105, Male)
		“They will say it’s for mental people only, it’s not for you, they are hard to believe that their children have mental issues” (106, Female)
Systemic/structural factors	Service availability	“I don’t think there is any specific services dedicated for adolescents” (102, Male)
		“There are very few psychiatrists and clinical psychologists working available and because of very few staff it also affect the services” (102, Male)
		“There is need of more specialists, but to study that subject [child and adolescent mental health] we have to go to India or abroad” (109, Male)
	Logistical	“Travel is an issue for geographical reasons, because if they are working in remote areas, they have limited access and transportation” (107, Female)
		“Those who are living around remote areas they have to allocate a day or two” (102, Male)
	Cost	“The adolescents don’t have much job opportunities in Nepal, and even if they want to get a job, because of the stigma they can’t…they have to rely on their parents for medical expenses” (103, Female)
		“Nepal’s healthcare is most cheap and easy than any countries that I have visited. It’s not about payment, it’s about the stigma” (105, Male)
		“They have to travel and take medicines regularly and there is some cost associated” (107, Female)

Individual Factors

Firstly, individual factors play a large role. Many participants stated that adolescents lack knowledge about mental health conditions and therefore do not realize when they have them. Participants also mentioned that adolescents do not have knowledge about the types of mental health services available.

Even if adolescents are aware of mental health services, misconceptions surrounding mental health treatment cause issues. Some believe that only medicines will cure them, and that counselling is ineffective.

Furthermore, one participant described how the suicide prevention hotline available at their health facility is misused due to a lack of knowledge about its purpose. An explanation for the lack of knowledge is lack of education in schools. The school syllabus does not contain mental health teaching.

Superstitious and religious beliefs were mentioned by three participants as a barrier, as adolescents believe that seeking help from alternative methods will better cure them. Furthermore, adolescents may be in denial about their mental health condition or wish to deal with it by themselves, preventing access.

Social Factors

The second theme was social factors. All participants stated that stigma is a barrier, with stigma from society being mentioned as the biggest form. However, this stigma is mostly not present for other physical illnesses, as they are accepted as normal. One participant also revealed that there is mental health stigma among medical staff. Two other participants explained that because of the stigma associated with mental health facilities, adolescents request alternative facilities to be treated.

Adolescents are fearful of the impact that their mental health diagnosis will have on future job opportunities, and marriage, which prevents access. Interestingly, it was all female participants who talked about the impact on marriage.

Two participants said that parents often misunderstand that their children have mental health conditions like depression, believing behavior is due to pubertal changes. Therefore, they do not think that their children should seek help from mental health services.

If parents do understand that their child has depression, they are ashamed and prevent their children from accessing services to avoid people finding out. One participant revealed that, sadly, this results in suicides.

Systemic/Structural Factors

The third main theme was related to structural factors. Most participants said there is a lack of mental health services, particularly in rural areas. Furthermore, many participants stated that there are no specific mental health services for adolescents, meaning they must access the same services as adults.

Many participants said that there is a lack of mental health staff due to a lack of training programs, which can affect the availability and delivery of mental health services. Furthermore, one participant explained that there are no opportunities for psychiatrists in Nepal to specialize in child or adolescent mental health.

Difficulties travelling to mental health services were mentioned by many participants. This is particularly an issue for adolescents living in remote areas who must spend lots of time travelling long distances to reach services.

Inability to afford mental health services is another barrier. Other associated costs from travel and medicines exacerbate the financial burden. Several participants stated that financial issues are a greater barrier for adolescents than adults, due to a lack of employment opportunities for them in Nepal, meaning they require parents to pay. Interestingly, this view was not unanimous, as two other participants reported that the cost of mental health treatment is not a barrier, because it is cheap compared to abroad.

Strategies to improve access

Two main themes, and further subthemes, were identified for strategies to improve access to mental health services (Table 4).

**Table 4:** Strategies to improve access: themes and subthemes

Themes	Subthemes
Increasing awareness of mental health and services	Social media
	Education in schools
	Community/societal awareness
Increasing service availability	Training staff
	Governmental changes
	Telepsychiatry

Quotes from the transcripts relating to the themes of strategies to improve access are presented in Table 5 to illustrate the key findings. Quotes were largely retained verbatim, including grammatical features and local phrasing. However, minor edits were made where necessary to clarify meaning, with any additions or alterations indicated by square brackets within the quotations.

**Table 5:** Strategies to improve access according to the participants In parentheses at the end of each quote are participant gender and a three-digit number corresponding to the participant's unique identifier.

Theme	Subtheme	Quotes
Increasing awareness of mental health and services	Social media	“Many adolescents have access to social media compared to previous decades, so they can provide the right kind of information that these kind of disorders are common, these kind of symptoms can happen, they are treatable conditions” (109, Male)
		“Yes, social media can play a good role in getting access to mental health services as well as raising awareness” (103, Female)
	Education in schools	“Government should include mental health education while making education strategies and whilst planning syllabus of school curriculum” (103, Female)
		“One is schooling education, from the beginning level. If they know about the symptoms, if they recognise that it is a mental health problem, then they will access services” (109, Male)
		“Tell them about what are the normal changes of adolescence and what are the changes due to mental health problems to distinguish between them, and tell them about mental health symptoms, services available” (108, Female)
	Community/societal awareness	“The community should be made aware that it’s very normal and people should not be ashamed of it” (103, Female)
		“It could be radio, leaflets and materials we distribute so they can get information from other channels and come themselves” (102, Male)
		“Posters, boards, that type of media-related awareness” (108, Female)
Increasing service availability	Training staff	“This community model of mental health providing service at the community is working very well, we are getting improved cases…it should be continued” (108, Female)
		“Training to health workers, training to FCHVs [female community health volunteers]…they should be trained because they are from the community” (107, Female)
		“I think learning never ends, I think we still need some more expertise and skills, we need that kind of trainings and skills” (102, Male)
	Governmental changes	“Government should be more involved and government should support us more and allocate budget for mental health services purely” (101, Female)
	Telepsychiatry	“Doctor will call them. They say their problems, what medicines is working or not and doctor will write prescription…they can show it to their local pharmacy and they can get their medicine” (106, Female)
		“They [adolescents] don’t have access to their internet, phones, so they have to ask from their parents, and they won’t allow it” (106, Female)

Increasing Awareness About Mental Health and Services

Social media was suggested by five participants as a platform to increase awareness about mental health among adolescents. In contrast, two participants stated that not many adolescents have access to social media. Although parents may have mobile phones, they will not give their children permission to use them, therefore limiting feasibility. The use of social media to promote suicide prevention was discussed by two participants, suggesting similar strategies should be developed to increase awareness. 

Education in schools about mental health is an important strategy to improve awareness, reduce stigma, and increase service access. Three participants suggested what should be taught, including normalizing mental health, explaining mental health symptoms, and outlining services which they can access. The appropriate ages to start delivery of mental health education, suggested by four participants, ranged from 10 to 14 years old.

Community awareness programs were recommended to normalize mental health and reduce associated shame and stigma, so that adolescents can access services without fear of stigma from the community. Alternative methods, such as radio, posters, and leaflets, were suggested by two participants as methods of raising awareness in society.

Increasing Service Availability

The importance of training community-level staff, like female community health volunteers, to increase community services was discussed. Training staff at the community level will reduce travel distances, time, and associated costs. Furthermore, staff from NGOs currently providing training described positive outcomes, so this strategy should be further implemented.

Despite the government developing a mental health strategy to increase inpatient beds, introduce mental health training for health workers, and provide free mental health medical expenses, it has not been implemented, so priority should be placed on ensuring this happens. Furthermore, two participants stated that mental health budget allocation should be increased to improve service availability and reduce costs for patients. 

Telepsychiatry is beneficial for patients in remote areas and could be used to increase services. However, one participant mentioned that this strategy might have limited success for adolescents with no access to a mobile phone or internet themselves.

## Discussion

Barriers to access

Lack of knowledge about mental health conditions and services was a prominent subtheme in this study. These findings are consistent with Wahid et al. [20], who found that some adolescents with depression in Nepal were unaware of their condition until their symptoms became severe, preventing access to help. In Malaysia, classified as an upper-middle-income country, 58% of adolescent participants with depression did not seek help because they thought that no treatment was available [21]. This demonstrates the importance of strategies to enhance adolescents’ mental health awareness and knowledge.

The most frequently reported barrier in this study was public stigma. Stigma is a strong disapproval that a group of people or members of a society hold about something [22]. It is well established that stigma delays access to mental health services among adolescents in both LMICs and high-income countries (HICs) [23,24]. One systematic review across multiple countries found that 76% of studies reported stigma as a barrier for children and adolescents in accessing mental health services [25]. Interestingly, although only mentioned by one participant in this study, another systematic review found that stigmatizing beliefs and attitudes were common among healthcare professionals in HICs and LMICs [26].

A study in Malaysia found that adolescents with depression with high levels of self-stigma have reduced help-seeking attitudes for mental health services [27]. Self-stigma, which is when a person applies these feelings of disapproval to themselves [22], was not mentioned in this study, although this might be due to a lack of insight among adolescents, as they were not interviewed.

Supporting the findings of this study, the cost of mental health treatment has been shown to be a barrier for adolescents in LMICs [28]. Although most participants in this study agreed that cost is a barrier, two participants disagreed. However, these participants disclosed experience of healthcare abroad, which might explain why they believed that it was not as costly in Nepal compared to other regions [29].

Lack of services, in combination with insufficient trained staff and specialized psychiatrists, was a commonly mentioned barrier in this study. Service improvement can be developed at two levels: firstly, by providing more basic healthcare services, generally provided by paramedics and general physicians [30], and secondly, in hospitals with specialized doctors [30]. The State of the World’s Children report found that the number of psychiatrists who specialize in child and adolescent mental health is fewer than 0.1 per 100,000 in all but HICs, demonstrating a lack of mental health staff worldwide [31]. In Nepal, there are only 0.01 per 100,000, suggesting a severe need to increase training opportunities [32].

Strategies to improve access

This study suggests that social media could be beneficial in promoting mental health awareness and reducing stigma. However, a different study found that social media was reported as a contributor to depression by almost all adolescent respondents in Nepal, for reasons such as social comparison and social withdrawal [20]. Therefore, research into the benefits and risks of using social media to promote mental health awareness in Nepal among adolescents should be considered.

Almost all participants in this study suggested school mental health education as a strategy to reduce barriers. A systematic review involving 22 studies from LMICs and HICs supports this, finding that school-based mental health programs (SBMHPs) increased knowledge and awareness of mental health disorders and the ability of adolescents to recognize signs and symptoms [33]. Furthermore, mental health stigma was reduced in six of the studies, demonstrating the efficacy of SBMHPs in reducing multiple barriers [33]. In Nepal, the “One School One Nurse” initiative was implemented in 2018/2019, with the aim of improving health outcomes of school children through education and healthcare [34]. Although the program is still in the process of being expanded, it provides an opportunity for SBMHPs to be facilitated within schools. Specifically, school nurses could be provided with mental health gatekeeper training to allow them to recognize early signs of depression and other mental health difficulties, provide basic support and education, and facilitate appropriate onward referral to mental health professionals if necessary, without requiring the development of an entirely separate service.

Telepsychiatry, which is a way of delivering mental healthcare via telecommunications technology like secure video conferencing [35], was only mentioned by one participant in this study. However, research has looked at the use of telepsychiatry in other LMICs for adolescents with depression where there are shortages of health professionals and infrastructure [35]. Other reported benefits include adolescents feeling more comfortable in their surroundings and being able to access mental healthcare without being seen by others and feeling discriminated against [36]. However, issues with technology costs and interrupted transmissions may limit success [37].

Training staff about mental health, particularly at the community level, was suggested in this study. A systematic review of psychological treatments delivered by health workers in primary and community settings in LMICs found moderate to strong effects in reducing mental health burden, demonstrating feasibility using this workforce [38]. However, Luitel et al. found that the effectiveness of training programs trialed in Chitwan, Nepal, was limited by the population’s knowledge about available services, further illustrating the need for awareness programs within schools and communities [39]. Furthermore, some health workers in Nepal are reluctant to take on extra work related to mental health service delivery due to a lack of incentives [40]. Therefore, this study suggests that, to ensure the successful delivery of mental health services at the community level, the government should consider providing incentives to encourage health workers to participate in and support these initiatives.

Strengths and limitations

One major strength of this study is its focus on the perspectives of mental healthcare professionals. As these professionals are directly involved in delivering mental health services, they have valuable insight into the barriers that can prevent adolescents from accessing treatment. Their viewpoint is also particularly important as they are better able to explain why existing services may be underutilized or unsuccessful, including organizational, workforce, and resource-related limitations, factors of which may not be readily recognized by service users themselves. 

This study has a context-specific focus in Pokhara, Nepal. A strength is that since barriers have been explored within this region specifically, findings are more directly relevant to the development of interventions and policies within this area. However, this can also act as a limitation, as barriers in other urban areas of Nepal were not explored. This therefore reduces generalizability to other urban contexts.

A limitation is that the quality of data collected might have been reduced since the lead researcher was inexperienced in conducting qualitative interviews and analysis. Furthermore, the interview guide was not pilot-tested, meaning the quality of initial interviews might have been reduced. 

Investigator and methodological triangulation were not implemented since only one researcher and one data collection method were used, reducing the credibility and validity of the research. However, time and resource constraints contributed to this.

Furthermore, another limitation is the use of purposive sampling. The organizations were identified and contacted through existing networks and contacts of the host organization, HERD International. This may have introduced selection bias, and the sample may not have captured the full range of perspectives or experiences of mental healthcare providers in Pokhara, Nepal, which may limit the transferability of the findings.

A methodological limitation is that interpreters were not professionally trained, and translation validity was not formally assessed, meaning technical and conceptual accuracy of translation might have been reduced. Furthermore, when interviews were conducted in English, there were some language barriers and misunderstanding of questions. Future studies could address these limitations by conducting all interviews in the native language and implementing techniques such as back-translation to reduce interpreter bias.

A final limitation relates to causality. In this study, healthcare professionals identified several factors that they believe contribute to reduced access to mental health services; however, the study cannot establish whether these factors directly cause reduced access.

Future research could explore barriers to accessing mental health services and potential strategies for overcoming these barriers in other urban areas of Nepal, as well as in rural communities, to overcome the limitation of using a single region. Furthermore, the findings relating to adolescents are based solely on the perspectives of healthcare providers, which may limit the credibility and comprehensiveness of the findings. Future studies could therefore incorporate a range of perspectives, including those of adolescents, their parents, and government staff, to develop a more comprehensive understanding of the barriers to accessing mental health services and the most appropriate strategies for addressing them in Nepal. 

Additionally, future research could investigate the feasibility and acceptability of telepsychiatry for adolescents in Nepal as a potential approach to improving access to mental health services, using interviews with adolescents, parents, and healthcare professionals to explore their experiences and perspectives.

## Conclusions

This study has identified multiple barriers to accessing mental health services for adolescents with depression in urban Nepal. Individual factors were mostly related to a lack of knowledge about mental health and available services. Social factors included stigma, largely from family and society, while systemic and structural factors focused on a lack of services and insufficient mental health staff. Strategies offered to overcome the most prevalent barriers include improving awareness about mental health within schools and through social media, and increasing the mental health workforce to provide these services within communities. Since many barriers are interlinked, multiple strategies must be developed and implemented simultaneously to maximize effectiveness. A holistic approach is necessary, involving cooperation between the government, NGOs, healthcare providers, and society.

## Appendices

**Table 6:** Interview guide

Subsections	Questions
Opening questions	Could you tell me about your job role?
	What type of facility do you work at?
	How long have you been in this role?
	How long have you worked with adolescents with depression?
To identify depression services available to adolescents in urban Nepal	What type of depression services are available for adolescents?
	What services does each facility offer?
	Where are depression facilities located?
	How do people access the service? Are they referred by someone? If so, who can refer? Can people also come directly to the service without a referral?
	Do adolescents have to pay for services?
	Are there any private depression services?
	Do you and your colleagues feel well trained to deliver depression care?
	What do you think is particularly good about the depression service you work at?
	Is there anything that would improve the service further?
To explore barriers to accessing depression services	What do you think are the biggest barriers to accessing depression services for adolescents?
	In your opinion, is there a difference between services available for adolescents and for adults?
	In your opinion, are there any barriers which are greater for adolescents than for adults when accessing depression services?
	Do you think there is a difference in depression services available in rural vs urban areas?
	Can adolescents attend alone, or do they have to come with parents?
	Does lack of knowledge about symptoms of depression affect access to services?
	Does lack of knowledge about available services affect access?
	Does stigma prevent patients attending? How does stigma impact on adolescents? How does stigma impact on their families?
	What methods of transport can people use to access services? Does this prevent access?
	Does availability of staff reduce access?
	Does cost prevent patients attending?
To identify strategies to reduce barriers to accessing depression services	What strategies could be introduced to overcome the barriers you mentioned to improve access to services?
	Are there any strategies in place now to improve access? (If yes, what are they?)
	Have any patients ever mentioned changes that they felt would help increase access to services?
